# Candidate biomarkers predicting clinical outcome after hyperbaric oxygen against chronic radiation cystitis

**DOI:** 10.1186/s12885-026-17309-z

**Published:** 2026-11-10

**Authors:** Daniel Giglio, Lana Sallam, Annika Thorsell, Johan Mölne, Nicklas Oscarsson, Helene Seeman-Lodding

**Affiliations:** 1 Department of Oncology, Institute of Clinical Sciences, Sahlgrenska Academy, University of Gothenburg, Gothenburg, Sweden; 2 Department of Medicine and Oncology, Southern Älvsborg Hospital, Borås, Sweden; 3 Proteomics Core Facility, Sahlgrenska Academy, University of Gothenburg, Gothenburg, Sweden; 4 Department of Clinical Pathology, Sahlgrenska University Hospital, Region Västra Götaland, Gothenburg, Sweden; 5 Institute of Biomedicine, Department of Laboratory Medicine, Sahlgrenska Academy, University of Gothenburg, Gothenburg, Sweden; 6 Department of Anaesthesiology and Intensive Care, Sahlgrenska University Hospital, Gothenburg, Sweden; 7 Department of Anaesthesiology and Intensive Care, Institute of Clinical Sciences, Sahlgrenska Academy, University of Gothenburg, Gothenburg, Sweden

**Keywords:** Radiation cystitis, Hyperbaric oxygen, Proteomics, Mast cells, Complement activation, Antigen presentation

## Abstract

**Background:**

Hyperbaric oxygen (HBO2) is effective against radiation cystitis. In this exploratory analysis we employed proteomics to assess biomarkers predicting clinical efficacy and the pharmacodynamics of HBO2 in radiation cystitis.

**Methods:**

Global quantitative proteomics was performed on biopsies taken before and after HBO2 in patients with radiation cystitis from the RICH-ART trial (NCT01659723). The proteome of HBO2 responders (R) was compared to non-responders (NR) and between R and NR at baseline. Immunohistochemistry was employed to assess the expression of potential biomarkers in urinary bladder biopsies. Biological processes were identified by using the STRING database.

**Results:**

Among proteins more expressed in R than NR at baseline were neutrophil defensin 1 and less expressed were chymase and mast cell carboxypeptidase A (CPA3). Among processes differentiating R from NR at baseline were processes regulating translation and complement activation. The top enriched biological processes in NR after HBO2 were processes regulating antigen processing and presentation. Only a few processes were affected by HBO2 in responders. No significant differences in the number of chymase+, CPA3 + and tryptase+ mast cells were observed in bladder specimens between R and NR before and after HBO2. The staining intensity of tryptase, chymase, CPA3 and complement factors C3, C4 and C8 were similar between R and NR and after HBO2.

**Conclusions:**

Multiple processes regulating the immune system, complement activation and translation in radiation cystitis are changed after HBO2. However, only a few processes were associated with the clinical efficacy of HBO2. The involvement of mast cells in the pathogenesis of radiation cystitis is suggested.

**Supplementary Information:**

The online version contains supplementary material available at https://doi.org/10.1186/s12885-026-17309-z.

## Introduction

Radiotherapy is an important tool in the oncological arsenal against solid tumors of the pelvic viscera. However, targeting tumors such as of the prostate gland, the uterine cervix, the endometrium, rectum and urinary bladder leads many times to acute adverse effects such as urinary bladder urgency and frequency, hematuria and bowel disturbances [1]. In some cases, these adverse effects may become chronic, which may lead to impairment of quality of life [2]. Radiotherapy leads to urothelial desquamation and barrier dysfunction of the urinary bladder and, further, impairs the normal tissue microvasculature [3]. Oxidative stress, antioxidation and inflammation occur in the irradiated urinary bladder [4–6]. However, the effects of radiotherapy on healthy tissue are complex and time-dependent and we previously showed that radiation may also inhibit the immune system of the urinary bladder [7]. Furthermore, we demonstrated that bladder irradiation may affect the micturition reflex and potentially affect bladder interstitial cells, thereby altering the neuronal control of the urinary bladder [8]. Altogether, radiotherapy changes the bladder function on several levels including altering the balance between oxidation and antioxidation, inflammation, neuronal control and urinary bladder tissue constitution.

Hyperbaric oxygen therapy (HBO2) is effective against radiation cystitis and may help patients even several years after the debut of symptoms [9]. However, not all patients with radiation cystitis are helped by HBO2. Clinical protocols of up to 40 sessions of HBO2 for radiation cystitis are presently used [9], which is time consuming for the patient and associated with high health care costs. At present, no biomarkers exist distinguishing patients benefitting from HBO2 from patients not benefitting from HBO2 against radiation cystitis and proctitis. The underlying pharmacodynamic mechanisms behind the clinical effects of HBO2 against radiation-induced side effects have been studied only sparsely. Moreover, only a few animal models have been developed. In an animal model for radiation-induced mucositis, HBO2 was shown to increase microvessel density in the oral mucosa [10]. Furthermore, HBO2 attenuated irradiation-induced changes of the murine mandible [11]. Oxidative stress and antioxidants were upregulated in the urinary bladder after irradiation in a rat model for radiation cystitis, where the upregulations of oxidative stress and antioxidants were attenuated by HBO2 [4].

Proteomic profiling has been employed for identifying prognostic biomarkers for urinary bladder cancer from biopsies and urinary samplings [12, 13]. Animal models show that complement and coagulation cascade processes are involved in the pathogenesis of radiation proctitis [14]. Few studies have assessed the pathogenesis of radiation cystitis and the effects of HBO2. Higher urine levels of hepatocyte growth factor (HGF) and placental growth factor (PlGF) were shown in radiation cystitis, and higher urine levels of vascular endothelial growth factor (VEGF) were demonstrated in patients with radiation cystitis with hematuria [15]. Plasma VEGF was also higher in patients with radiation cystitis before HBO2 compared to patients undergoing HBO2 for other causes than radiation cystitis [16].

Given the current lack of biomarkers capable of predicting the clinical efficacy of HBO2 against radiation cystitis we aimed in this exploratory study to identify potential biomarkers associated with the therapeutic response of HBO2. We employed global quantitative proteomics and immunohistochemical analysis to characterize the proteomic profiles of patients with radiation cystitis who responded to HBO2 versus those who did not. Moreover, we investigated the biological processes distinguishing HBO2 responders from non-responders and assessed physiological changes following HBOT to elucidate mechanisms underlying therapeutic benefit.

## Methods and Materials

### Patient cohort

The patient cohort analyzed is constituted by a sub-group of patients with radiation cystitis undergoing HBO2 from the RICH-ART trial [9]. We here selected the four patients with the best clinical outcome, i.e., the best increase in points from baseline till follow-up on the Expanded Prostate Cancer Index Composite (EPIC)-urinary domain and four patients with the worst clinical outcome on the EPIC-urinary domain for proteomics on paraffin-embedded urinary bladder biopsies taken prior to HBO2 and 4–6 months after HBO2 (on the same day as completion of the EPIC questionnaires). The median age of the 8-patient cohort was 65 years (range, 29–72). Patients were dichotomously grouped as “responders” vs. “non-responders” to HBO2 depending on the change in urinary EPIC score (improvement of ≥ 9 points was considered a clinically relevant change). Patients were not treated with anti-inflammatory drugs or antioxidants that might influence the proteomic profile. Immunohistochemistry was performed on bladder paraffin-embedded biopsies taken from 12 patients responding to HBO2 (≥ 9 points improvement on the urinary EPIC score) and 8 patients not responding to HBO2 (< 9 points improvement on the urinary EPIC score). The demographics are displayed in Table 1.

**Table 1:** Demographics of the cohort

Patient 1	Gender	Age at baseline	Cancer type	EPIC-uro at baseline	EPIC-uro at follow-up	Delta EPIC	HBO2 Response	Proteomics
	Male	71	Rectal	18.1	91.7	73.6	Yes	Yes
**Patient 2**	Male	64	Prostate	39.6	82.7	43.1	Yes	Yes
**Patient 3**	Male	68	Prostate	34.8	95.8	61.0	Yes	Yes
**Patient 4**	Female	48	Cervical	39.6	80.6	41.0	Yes	Yes
**Patient 5**	Female	70	Other (C44.5)	29.8	38.2	8.4	No	Yes
**Patient 6**	Female	32	Cervical	25.7	23.6	-2.1	No	Yes
**Patient 7**	Female	29	Cervical	40.3	48.6	8.3	No	Yes
**Patient 8**	Female	62	Cervical	55.5	56.9	1.4	No	Yes
**Patient 9**	Female	38	Cervical	50.0	78.5	28.5	Yes	No
**Patient 10**	Male	66	Prostate	57.7	89.6	31.9	Yes	No
**Patient 11**	Female	35	Cervical	66.7	95.8	29.1	Yes	No
**Patient 12**	Male	80	Prostate	74.3	87.5	13.2	Yes	No
**Patient 13**	Male	64	Prostate	72.3	84.8	12.5	Yes	No
**Patient 14**	Female	45	Cervical	45.2	74.3	29.1	Yes	No
**Patient 15**	Female	44	Cervical	72.9	97.3	24.4	Yes	No
**Patient 19**	Female	41	Cervical	43.8	59.1	15.3	Yes	No
**Patient 16**	Male	68	Prostate	21.5	25.7	4.2	No	No
**Patient 17**	Male	67	Prostate	50.0	56.3	6.3	No	No
**Patient 18**	Female	72	Cervical	53.5	53.5	0	No	No
**Patient 20**	Male	78	Prostate	29.8	38.2	8.4	No	No

### Global relative quantification

Details for the experiment are provided in Supplementary methods. Relative quantification was performed to compare protein expression in deparaffinized formalin-fixed paraffin-embedded urinary biopsies (20 sections à 10 μm). Proteins were extracted in 2% sodium dodecyl sulfate lysis buffer using a FastPrep instrument (Matrix D, MP Biomedicals, Irvine, CA, USA), processed and digested using the modified filter-aided sample preparation (FASP) method. Peptide samples were labelled using TMTpro 16-plex isobaric mass tagging reagents (Thermo Fisher Scientific, Waltham, MA, USA), pooled into one TMT-set, purified by Pierce Peptide Desalting Spin Columns. The TMT-set was basic reversed-phase chromatography (bRP-LC, pH 10) fractionated into 20 fractions over 30 min. Each fraction was analysed on Orbitrap Fusion Tribrid mass spectrometer interfaced with nLC 1200 liquid chromatography system (both Thermo Fisher Scientific). Peptides were separated on a C18 35 cm column over 90 min and data were acquired with the SPS MS3 method. Raw files were processed and analysed with Proteome Discoverer (ver 2.4, Thermo Fisher Scientific) against UniProt Swiss-Prot Human using Mascot as a search engine. The MS proteomics data have been deposited to the ProteomeXchange Consortium (http://proteomecentral.proteomexchange.org) via the PRIDE partner with the data set identifier PXD085707 [17].

### Immunohistochemistry

For protocol 1 (chymase, CPA3 and tryptase), tissue sections were stained using the Autostainer Link 48 (Agilent Dako, Santa Clara, CA, USA). The sections were first hydrated in decreasing concentrations of ethanol and deparaffinized in xylene. Tissue sections were then bathed in PT-link for 20 min (max 97℃) for antigen retrieval followed by incubation with peroxidase block for 5 min and washes in Tris-buffered saline-Tween 20 (TBS-T: pH 7.6). The sections were then incubated with HRP for 20 min followed by DAB staining for 10 min. Hematoxylin staining was then performed and the sections were washed in dH_2_0 and cover glasses were mounted on top of the sections. For protocol 2 (C3, C4b and C8a) immunostainings were performed manually. After hydration in decreasing concentrations of ethanol and deparaffinization in xylene, tissue sections were boiled in citrate buffer or ethylenediaminetetraacetic acid (EDTA) solution for antigen retrieval. The tissue sections were incubated with hydrogen peroxide for 30 min, washed in phosphate-buffered saline (PBS) and then blocked with goat serum for 30 min followed by an overnight incubation with the primary antibody overnight at 4℃. The next day, the sections were incubated with the secondary antibody for 1 h followed by DAB staining for 10 min. Hematoxylin was added to the sections, which were then rinsed with running tap water. The sections were dehydrated in increasing concentrations of ethanol and cover glasses were then placed on the sections with Pertex mounting medium (Histolab, Gothenburg, Sweden).

### List of primary and secondary antibodies

Goat anti-rabbit IgG (HRP) (1:500, Abcam, ab6721, Cambridge, MA, USA), mouse anti-tryptase (1:5000; ab2378, Abcam), rabbit anti-CPA3 (1:500; ab251685, Abcam), rabbit anti-mast cell chymase (1: 500; ab233729, Abcam), rabbit anti-C4b (1:50; ab181241, Abcam), rabbit anti-C3 (1:2000; ab200999, Abcam) and rabbit anti-C8a (1:200; PA5-144217, Invitrogen).

### Assessment of immunohistochemistry

The assessment of staining intensity and the number of chymase, CPA3 and counting tryptase-positive cells were performed blindly. Stained sections were scanned with the Nanozoomer S210 Digital Slide Scanner (Hamamatsu Photonics K.K., Shizuoka, Japan) and the number of positive cells and the calculation of surface area were performed using the NIS-Elements software (Nikon, Inc., Melville, NY, USA). The staining intensity of mast cell markers (chymase, tryptase and CPA3) and complement factors (C3, C4b and C8a) were analyzed in each bladder biopsy. An intensity score between 0 and 3 was used, where 0 indicates no expression, 1 indicates low expression, 2 indicates moderate expression and 3 indicates high expression.

### Data analysis and statistics

The proteomes at baseline before and after HBO2 were compared in the responder and the non-responder groups to assess the proteome response to HBO2 in the two groups. The proteomes at baseline between responders and non-responders were also compared assessing whether baseline factors explained changes in clinical outcome to HBO2. Finally, the difference in response of HBO2 in responders and non-responders was compared to assess for differently expressed proteins (DEPs) and biological pathways correlated with the clinical efficacy of HBO2. The following comparisons of proteomes were made:

Comparison A. Responders and non-responders at baseline.

Comparison B. Non-responders before and after HBO2.

Comparison C. Responders before and after HBO2.

Comparison D. (After - before HBO2) in responders - (after – before) in non-responders.

To identify DEPs, Welch’s t-test on log2-transformed data for comparisons between independent groups, while a paired t-test was used for comparisons before and after HBO2 treatment. Proteins with a *p* < 0.05 were considered as differentially expressed. Given the small sample size, unadjusted p-values were used without correction for multiple testing. Proteins with an unadjusted p-value < 0.05 were considered differentially expressed. No fold-change threshold was applied to define DEPs; instead, fold change was used as a complementary measure of effect size and, together with the p-value, to rank DEPs and select proteins for subsequent analyses.

Fold change (FC) was calculated by dividing the the mean protein abundance for the non-responder group by that for the responder group. Volcano plots were used to visualize expressional differences between groups. To identify candidate biomarkers, we ranked proteins according to their Euclidean distance from the origin of the volcano plot, calculated as by the following equation √ (log2 fold-change^2^ + -log10 significance^2^), with proteins exhibiting the greatest distances considered to have the largest differences between groups.

For comparison A, B, C and D, the top 50 most changed proteins were considered. This selection corresponded to absolute fold-change cut-offs of ≥ 0.60, ≥ 0.45, ≥ 0.28 and ≥ 0.45 for comparisons A, B, C, and D, respectively. Due to the high heterogeneity among responder samples, an additional post-hoc analysis was performed using an absolute fold-change cut-off of ≥ 0.45 and an unadjusted p-value threshold of < 0.10. As an exploratory sensitivity analysis, enrichment analysis was also performed using all DEPs (*p* < 0.05) without applying a fold-change cut-off to assess whether the identified biological processes were robust to the selection of the top-ranked proteins.

We employed STRING (v.11.5; http://string-db.org/cgi/input.pl) using the Markov clustering algorithm to construct protein–protein interaction (PPI) networks based on all differentially expressed proteins (DEPs; both up- and downregulated), for the 50 most changed proteins (both up- and downregulated), for all upregulated DEPs or for all downregulated DEPs in each respective patient group. Heatmaps and PCA-plot were generated in Perseus (v2.0.7.0) and volcano plots in GraphPad Prism 10.5.0 (GraphPad Software, Inc., San Diego, USA). The Venn diagram was generated via the online tool available at https://bioinformatics.psb.ugent.be/webtools/Venn/.

For immunohistochemical data, the Kruskal Wallis test followed by the Dunn´s multiple comparison test was used to compare statistical significance between groups. Statistical significance was considered p-values < 0.05. The GraphPad Prism program 10.2.3 (GraphPad Software, Inc., San Diego, USA) was used for statistical analyses.

## Results

### Protein expressions at baseline and after HBO2

Global proteomic analysis quantified a total of 4,352 proteins. To assess overall proteomic variation, principal component analysis (PCA) was performed on all quantified proteins. As expected, samples from non-responders at baseline and after HBOT clustered more closely, indicating similar expression and small proteomic changes, whereas responder samples displayed greater heterogeneity. In contrast, the PCA of the individual patient ratios calculated by dividing after HBO2 with baseline indicated a separation of the non-responder and the responder (Supplementary Fig. 1).

**Fig. 1:**
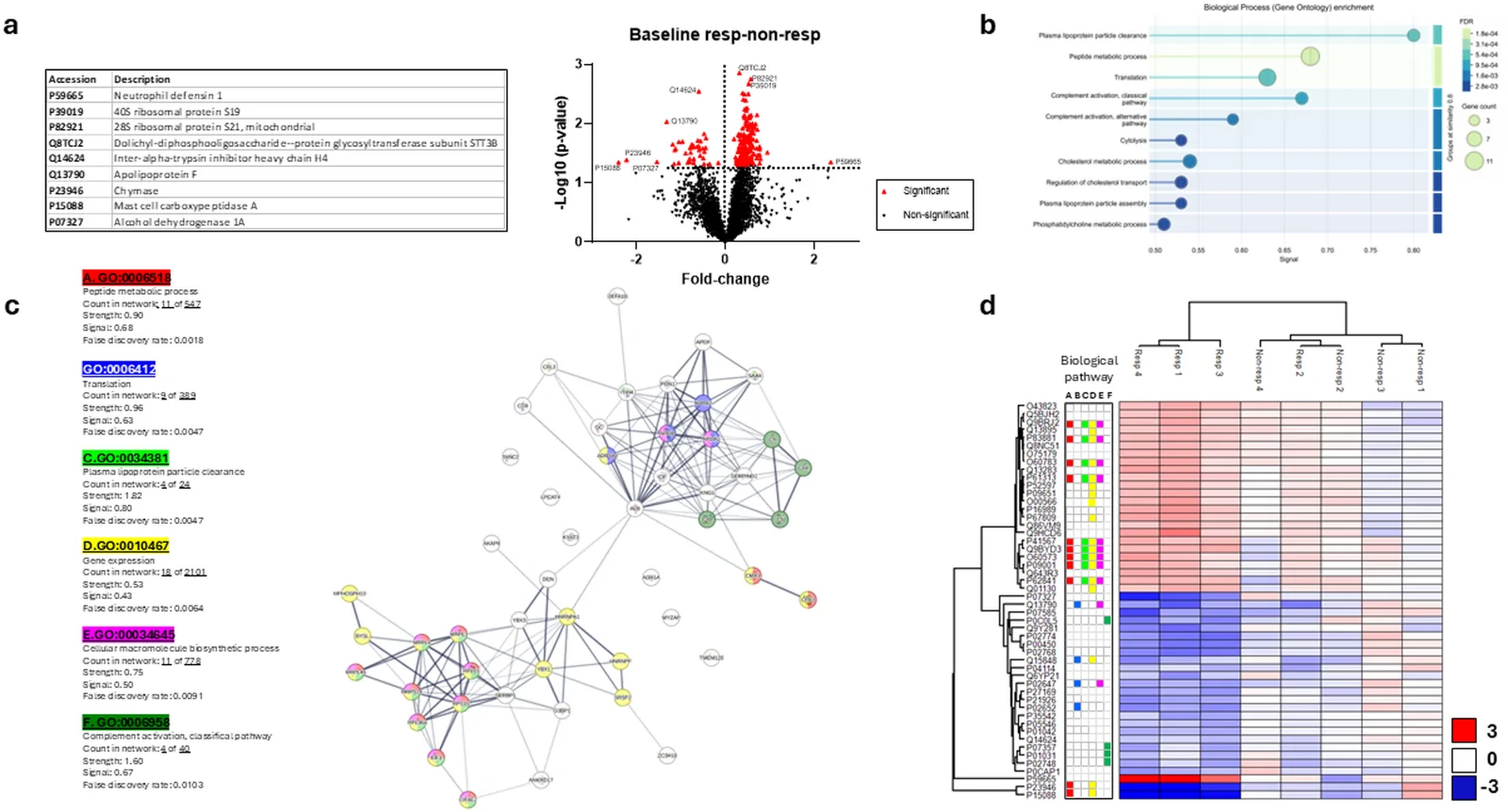
Urinary bladder proteins with both high fold-change and high significant difference between responders and non-responders at baseline before hyperbaric oxygen therapy (i.e., distant proteins from the origo of the volcano plot) and volcano plot depicting log2 fold-change vs. -log10 significance of the expression of baseline proteins for responders vs. non-responders at baseline (**a**); The top enriched biological processes for responders vs. non-responders at baseline considering the 50 most differently expressed proteins (**b**-**c**); Protein-protein network analysis (**c**) and heatmap (**d**) of the 50 most differently expressed proteins between the responder and non-responder groups at baseline

**Fig. 2:**
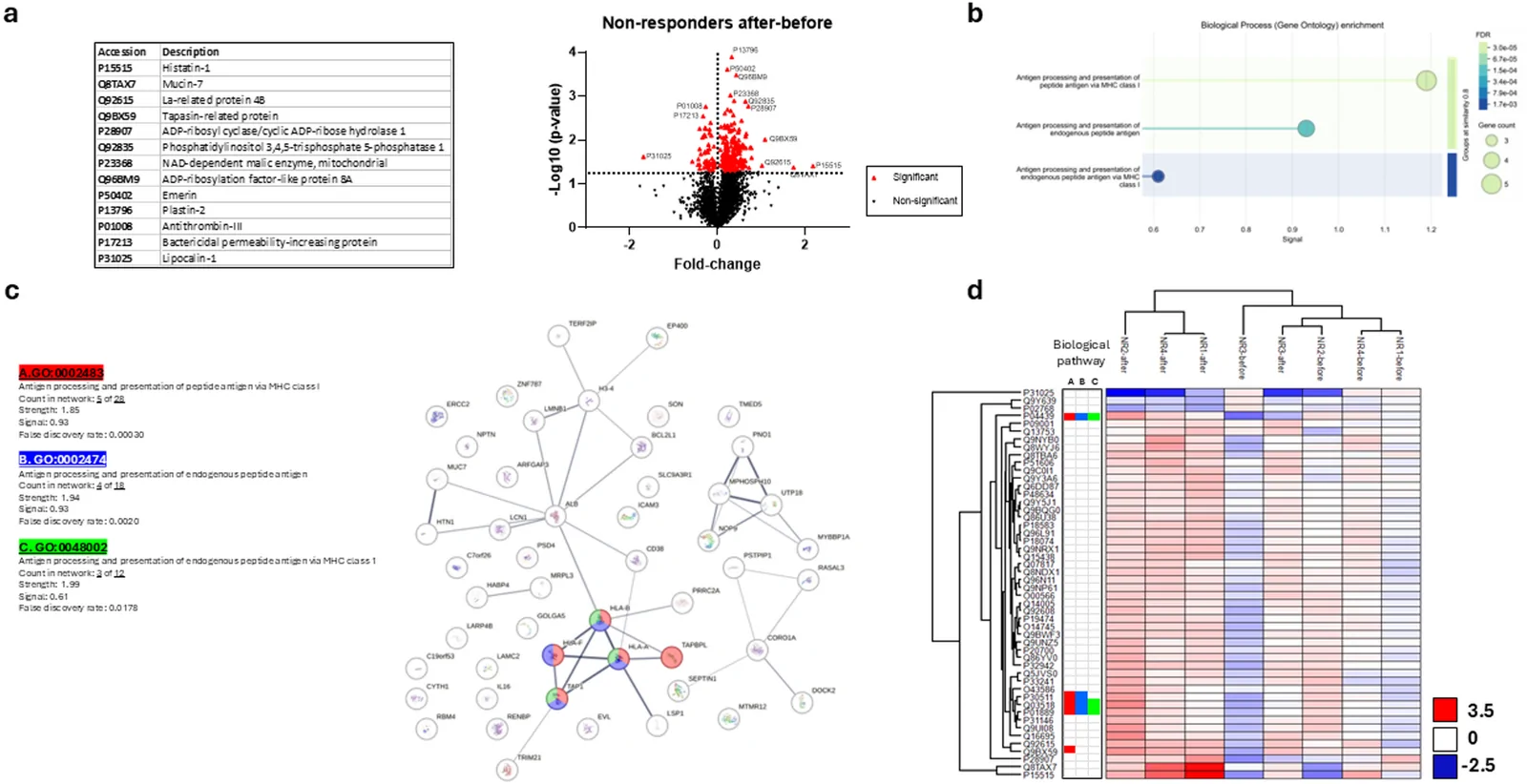
Urinary bladder proteins with both high fold-change and high significant change after hyperbaric oxygen (HBO2) in non-responders (i.e., distant proteins from the origo of the volcano plot) and volcano plot depicting log2 fold-change vs. -log10 significance of the expression of proteins after HBO2 in non-responders (**a**); The top enriched biological processes after HBO2 in non-responders considering the 50 most differently expressed proteins (**b**-**c**); Protein-protein network analysis (**c**) and heatmap (**d**) of the 50 most differently expressed proteins after HBO2 in non-responders

**Fig. 3:**
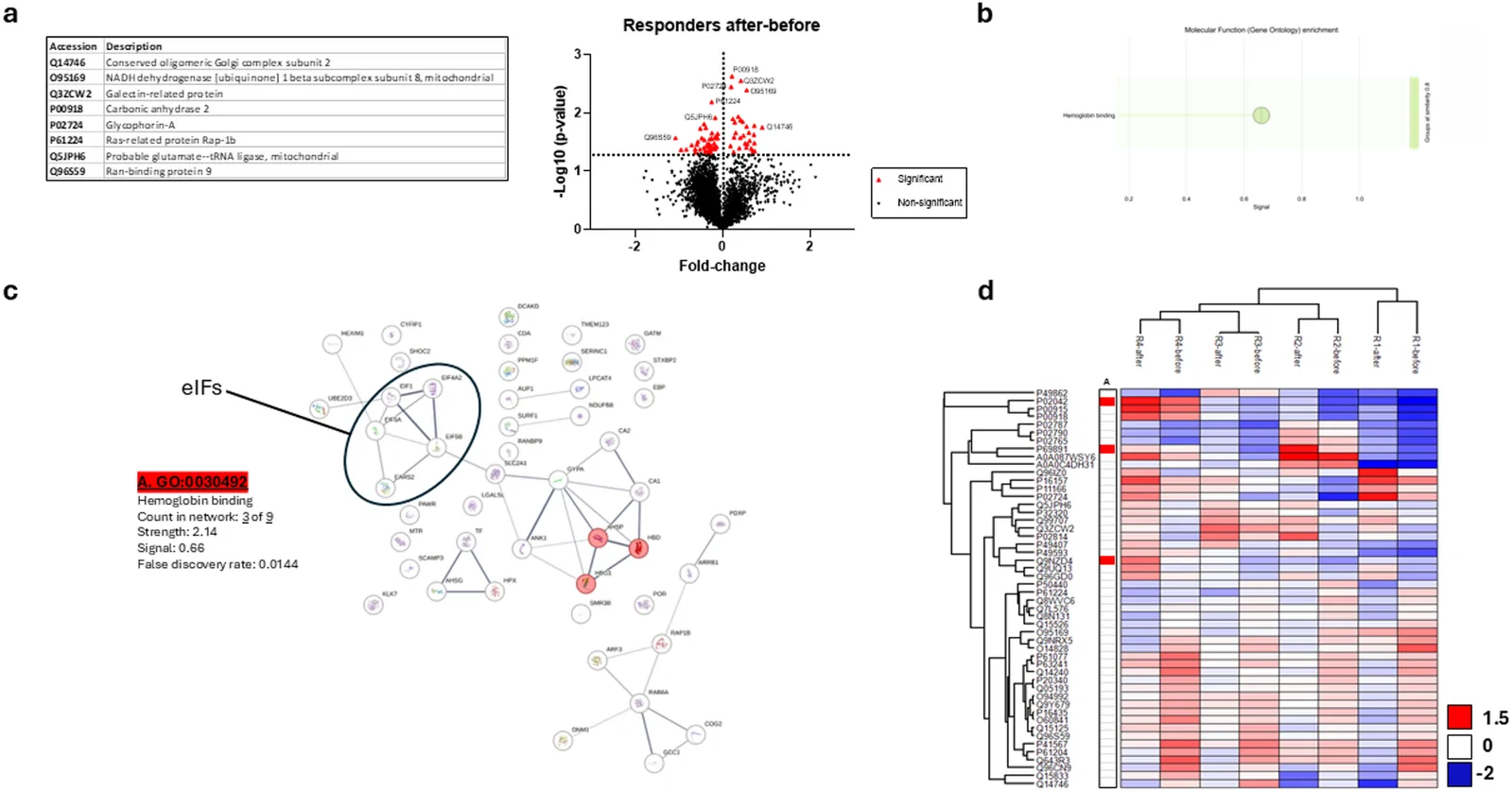
Urinary bladder proteins with both high fold-change and high significant change after hyperbaric oxygen (HBO2) in responders (i.e., distant proteins from the origo of the volcano plot) and volcano plot depicting log2 fold-change vs. -log10 significance of the expression of proteins after HBO2 in responders (**a**); The top enriched biological pathways after HBO2 in responders considering the 50 most differently expressed proteins (**b**-**c**); Protein-protein network analysis (**c**) and heatmap (**d**) of the 50 most differently expressed proteins after HBO2 in responders. Immunoglobulin kappa variable 3D-15 and Immunoglobulin heavy variable 1–18 were not found in the String database. eIFs=eukaryotic initiation factors

At baseline, 187 DEPs were more expressed and 38 DEPs less expressed in HBO2 responders compared to non-responders (Fig. 1a; comparison A; Supplementary Table​ 1​). In the non-responder group, 231 DEPs were upregulated and 51 DEPs were downregulated after HBO2 (Fig. 2a; comparison B; Supplementary Table​ 1​). In contrast, in the responder group, only 29 DEPs were upregulated and 44 DEPs were downregulated after HBO2 due to the higher heterogeneity in the samples (Fig. 3a; comparison C; Supplementary Table​ 1​). For comparison D, 24 DEPs were upregulated and 173 DEPs were downregulated (Supplementary Table 1​). The DEPs positioned the most distant from the origo of the volcano plots, i.e., distinguishing the groups the most from each other, are displayed in Figs. 1a, 2a and 3a. Interestingly, 45 DEPs were shared in the comparisons A (baseline responders vs. non-responders) and comparison B (non-responders baseline and follow-up). In contrast, only two DEPs were the same in comparison B and comparison C (responders baseline and follow-up) and 6 DEPs were the same in comparison A and comparison C (Supplementary Fig. 2).

### Pathways differentiating responders from non-responders at baseline

Among the most regulated DEPs the most distant from the origo of the volcano plot (i.e., both highly significant and highly changed) distinguishing the most responders from non-responders at baseline were neutrophil defensin 1 (*DEFA1*), 40 S ribosomal protein S19 (*RPS19*), 28 S ribosomal protein S21 mitochondrial (*MRPS21*), dolichyl-diphosphooligosaccharide–protein glycosyltransferase subunit STT3B (*STT3B*), inter-alpha-trypsin inhibitor heavy chain H4 (*ITIH4*), apolipoprotein F (*APOF*), chymase (*CMA1*), mast cell carboxypeptidase A (*CPA3*) and alcohol dehydrogenase 1A (*ADH1A*; Fig. 1a; for full list see Supplementary Table​ 1​).

Among the top 50 DEPs (up- and downregulated) between responders and non-responders (absolute fold-change ≥ 0.60), Peptide metabolic process, Translation, Plasma lipoprotein particle clearance, Gene expression, Cellular macromolecule biosynthetic process and Complement activation, classical pathway, were identified as enriched biological processes (Fig. 1b-d; Supplementary Table 2).

As an exploratory sensitivity analysis, enrichment analysis was also performed using all DEPs (*p* < 0.05) without applying a fold-change cut-off. This analysis identified largely overlapping biological processes, including Translation, Gene expression, Cytoplasmic translation, Peptide metabolic process, Cellular macromolecule biosynthetic process and Cellular amide metabolic process (Supplementary Fig. 3 and Supplementary Table 3). Proteins involved in translation showed significantly higher abundance at baseline in responders than in non-responders, whereas proteins involved in complement activation showed significantly higher abundance in non-responders than in responders (Supplementary Figs. 4–5 and Supplementary Tables 4–5).

### Pathways affected by HBO2 in non-responders

In the top of DEPs the most distant from the origo of the volcano plot (i.e., both highly significant and highly changed) were histatin-1 (*HTN1*), mucin-7 (*MUC7*), La-related protein 4B (*LARP4B*), tapasin-related protein (*TAPBPL*), ADP-ribosyl cyclase/cyclic ADP-ribose hydrolase 1 (*CD38*), phosphatidylinositol 3,4,5-trisphosphate 5-phosphatase 1 (*INPP5D*), NAD-dependent malic enzyme mitochondrial (*ME2*), ADP-ribosylation factor-like protein 8 A (*ARL8A*), emerin (*EMD*), plastin-2 (*LCP1*), antithrombin-III (*SERPINC1*), bactericidal permeability-increasing protein (*BPI*) and lipocalin-1 (*LCN1*; Fig. 2a; for full list see Supplementary Table​ 1​).

Considering solely the top 50 DEPs (absolute fold-change ≥ 0.45), Antigen processing and presentation of peptide antigen via MHC class I, Antigen processing and presentation of endogenous peptide antigen and Antigen processing and presentation of endogenous peptide antigen via MHC class 1 were identified as enriched biological processes (Fig. 2c-d; Supplementary Table 6). Among upregulated DEPs were coronin-1 A and RAS protein activator like-3 (Supplementary Table​ 1​). Among downregulated DEPs were the antioxidants superoxide dismutase-1 (SOD-1) and catalase (Supplementary Table​ 1​).

Enrichment analysis of all DEPs (*p* < 0.05) without applying a fold-change cut-off identified Gene expression, RNA processing, Cellular nitrogen compound metabolic process, Cytoplasmic translation, Peptide metabolic process and RNA splicing as enriched biological processes (Supplementary Table 7). Including only upregulated DEPs in the STRING protein-protein interactions database, identified the same top enriched biological processes as including all DEPs (Supplementary Fig. 6; Supplementary Table 8). The enriched biological processes considering only downregulated DEPs after HBO2 were Response to toxic substance and Response to stimuli (Supplementary Fig. 7; Supplementary Table 9).

### Pathways affected by HBO2 in responders

For responders to HBO2, among the DEPs the most distant from the origo of the volcano plot (i.e., both highly significant and highly changed by HBO2) we identified Conserved oligomeric Golgi complex subunit 2 (*COG2*), NADH dehydrogenase [ubiquinone] 1 beta subcomplex subunit 8 mitochondrial (*NDUFB8*), galectin-related protein (*LGALSL*), carbonic anhydrase 2 (*CA2*), glycophorin-A (*GYPA*), Ras-related protein Rap-1b (*RAP1B*), probable glutamate–tRNA ligase mitochondrial (*EARS2*) and ran-binding protein 9 (*RANBP9*; Fig. 3 a; for full list see Supplementary Table​ 1​).

Including the top 50 DEPs (absolute fold change ≥ 0.28; *p* < 0.05) or all 73 DEPs in the STRING protein-protein interactions database, only the molecular pathway Hemoglobin binding was enriched (Fig. 3b-c and Supplementary Table 10, respectively). To note, eukaryotic initiation factor (EIF)1, EIF5A, EIF5B and EIF4A2 were downregulated (fold-change 0.24–0.62) after HBO2 in responders (Fig. 3c and Supplementary Table​ 1​).

Due to large variances in the responder group (see Supplementary Fig. 1), we also included all 90 DEPs with an absolute fold change of ≥ 0.45 and with a p-value of < 0.10 in the String analysis protein-protein interaction database showing that Cytoplasmic translation, Carbon dioxide transport, Peptide biosynthetic process, Translation, Peptide metabolic process, Hydrogen peroxide catabolic process and Regulation of translational initiation were significantly enriched biological pathways (Supplementary Fig. 8 and Supplementary Table 11).

### Pathways differently changed after HBO2 between responders and non-responders

In the volcano plot of DEPs after HB02, comparing responders and non-responders, the most distant point from the origo was lipocalin-1 (*LCN1*) followed by mammaglobin-B (*SCGB2A1*), albumin (*Alb*), GRIP and coiled-coil domain-containing protein 1 (*GCC1*), cytochrome b-245 light chain (*CYBA*), golgin subfamily A member 5 (*GOLGA5*) and tapasin-related protein (*TAPBPL*; Supplementary Fig. 9; for full list see Supplementary Table 1​). Lipocalin-1, mammaglobin-B and albumin were numerically downregulated after HB02 in responders and numerically upregulated in responders, while the opposite occurred for GRIP and coiled-coil domain-containing protein 1, cytochrome b-245 light chain, golgin subfamily A member 5 and tapasin-related protein.

Among biological processes, only carbon dioxide transport was enriched when the top 50 DEPs (absolute fold change ≥ 0.89; *p* < 0.05) were included (Supplementary Table 12). In contrast, analysis of the top 173 DEPs (absolute fold change ≥ 0.45; *p* < 0.05) identified Translation, Cytoplasmic translation, Peptide metabolic process, Stress granule assembly, Cellular macromolecule biosynthetic process, and Cellular amide metabolic process as the most enriched biological processes. Many of the enriched biological pathways overlapped with the enriched biological pathways at baseline between responders and non-responders (Supplementary Fig. 9 and Supplementary Table 13​).

### Immunohistochemical assessment of mast cells and complement factors

**Fig. 4:**
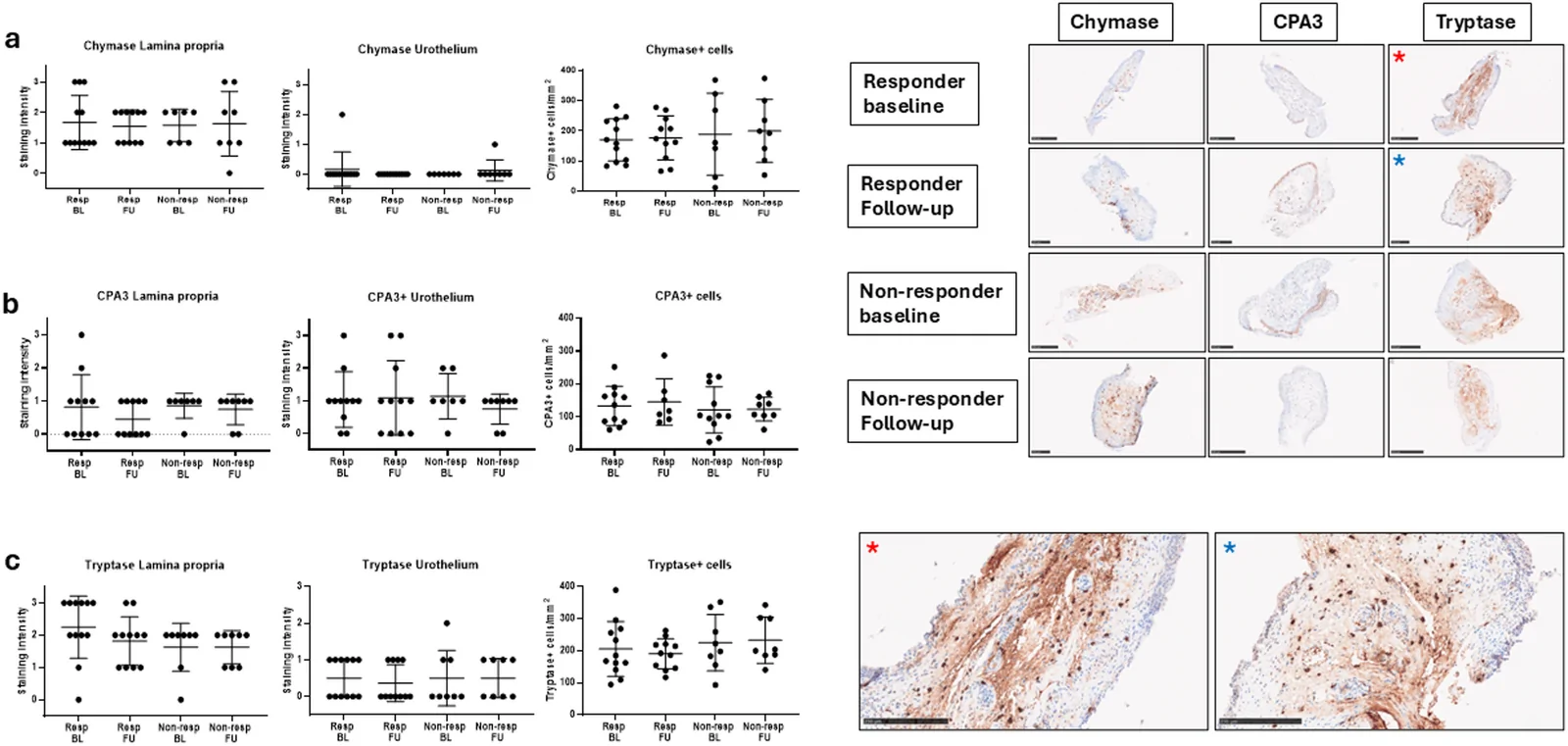
Photomicrographs of urinary bladder biopsies taken from non-responders and responders before and after hyperbaric oxygen (HBO2) and stained for the expression of chymase (**a**), carboxypeptidase A (CPA3; **b**) and tryptase (**c**) in the lamina propria and urothelium and total number of chymase+, CPA3 + and tryptase+ cells in the lamina propria and urothelium/mm^2^. Counterstaining with hematoxylin. Scale bars = 250–500 μm

No microscopical changes were observed between responders and non-responders or after HBO2 in tissue specimens. Since chymase and CPA3, both important enzymes for mast cell function, were significantly more expressed in non-responders than in responders before HBO2, we assessed the involvement of mast cells in the development of radiation cystitis and treatment efficacy of HBO2. Urinary bladder tissue specimens were therefore stained against chymase, CPA3 and tryptase (another important protease of mast cells). Chymase+, CPA3 + and tryptase+ mast cells were observed in the lamina propria of the urinary bladder specimens, however, no differences in the numbers of positive cells or in the staining intensity of chymase, CPA3 or tryptase were observed between responders and non-responders at baseline or after HBO2 (Fig. 4).

**Fig. 5:**
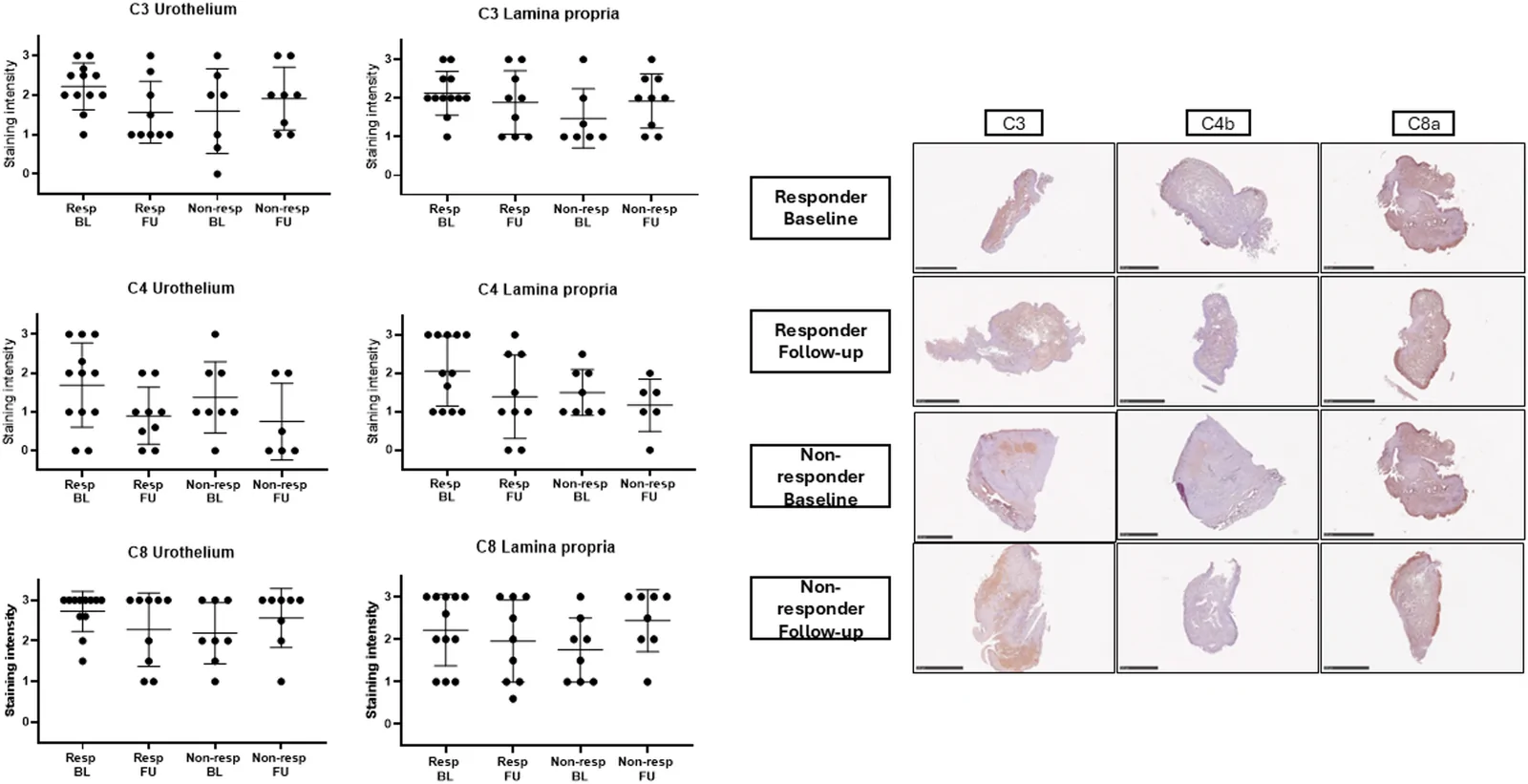
Photomicrographs of urinary bladder biopsies taken from non-responders and responders before and after hyperbaric oxygen (HBO2) and stained for the expression of complement C3 (**a**), complement C4 (**b**) and complement C8 (**c**) in the lamina propria and urothelium. Counterstaining with hematoxylin. Scale bars = 250–500 μm

Proteomics analysis showed that complement activation was enriched in non-responders compared to responders at baseline. To assess differences in the expression of the complement factors C3, C4b and C8a, immunostaining was performed on urinary bladder tissue obtained from responders and non-responders before and after HBO2. No significant differences were observed between responders and non-responders at baseline or after HBO2 (Fig. 5).

## Discussion

HBO2 is in many cases an effective treatment against adverse effects to radiotherapy but at the same time it is a time-consuming and costly treatment option. At present no biomarkers exist to identify patients that are helped by HBO2. In our exploratory analysis, with proteomics and immunohistochemistry, we presently identified potential biological processes and biomarkers of importance predicting clinical efficacy of HBO2 and biological processes activated by HBO2 in the irradiated urinary bladder.

### Involvement of mast cell enzymes in responses to HBO2

Neutrophil defensin 1, chymase and CPA3 were identified as potential biomarkers associated with response to HBO2 in patients with radiation cystitis. Previous studies have shown that human alpha defensin 1 constitutes a molecular brake on macrophage-driven inflammation by inhibiting macrophage mRNA translation [18]. Several clinical studies have evaluated the anti-inflammatory effects of brilacidin, a defensin-mimetic molecule, against various conditions including oral mucositis in patients with head and neck cancer receiving chemoradiotherapy [19]. Ventura *et*. *al*. demonstrated using proteomics that the abundance of neutrophil defensin 1 in the acquired enamel pellicle decreased in patients receiving radiotherapy for head and neck cancer [20]. The same study also reported a decrease in histatin-1 following radiotherapy. Interestingly, we observed that histatin-1 was among the most increased proteins after HBO2 in non-responders. Histatin-1 is involved in wound healing by stimulating fibroblasts in the murine skin [21]. Histatin-1 may therefore potentially contribute to tissue repair in the irradiated urinary bladder, although its functional role in this context remains to be established.

The differences in chymase and CPA3 abundance associated with the response to HBO2 suggest that mast cells may be involved in the pathogenesis of radiation cystitis. Hyperoxia may affect distribution and activity of mast cells, as demonstrated in an animal model of oxygen-induced retinopathy and in rat lungs exposed to hyperoxia [22–24]. The involvement of mast cells in normal tissue responses to radiation has also been shown in many studies. For example, radiation of the skin increases the expression of chymase and tryptase in the skin [25]. Radiation of the gut decreases the number of mast cells in the gut mucosa, and radiation proctitis is associated with the activation of chymase-positive mast cells [26]. Mast cells have also been implicated in urinary bladder disease such as interstitial cystitis/painful bladder syndrome and in cyclophosphamide-induced cystitis [27, 28].

However, in our study, immunohistochemical analyses did not reveal differences in the expression of chymase, CPA3, or tryptase between responders and non-responders. Neither were differences observed in the numbers of chymase-, CPA3-, or tryptase-positive mast cells between the groups. To note, both among responders and non-responders to HBO2 the variability in the number of mast cells present in the bladder mucosa was large. This variability, together with the lack of differences between groups in the immunohistochemical analyses, warrants caution when interpreting the proteomic findings and suggests that further studies are needed to clarify the role of mast cells in radiation cystitis and the response to HBO2.

### Translation and complement activation important biological processes

Proteins regulating translation were significantly more expressed in responders than non-responders, while proteins regulating complement activation were significantly more expressed in non-responders than responders. Complement activation by radiotherapy has been reported by many studies. To exemplify, complement factor C3 seems to be involved in the inflammatory response following the acute phase after radiation of the murine brain [29]. Complement factors C3 and C4 have been suggested to activated in the clearance of apoptotic immune cells of the murine spleen after whole-body irradiation [30]. In addition, complement factors are involved in the tumor-specific immune response following radiotherapy [31]. Evidence of complement involvement has also been reported in bladder disorders, e.g., in patients with interstitial cystitis the level of complement C3d is increased [32], while activation of the C5a/C5aR1 axis has been demonstrated in a murine model of acute bacterial cystitis [33].

Our findings may tentatively indicate that there are different categories of radiation cystitis where one category is driven by inflammation involving complement activation that is less sensitive to HBO2. The other category constitutes radiation cystitis with a lower inflammatory drive more responsive to HBO2. We could, however, not discern expressional differences in C3, C4b or C8a in bladder tissue with immunohistochemistry between responders and non-responders, either at baseline or after HBO2. This is most likely due to large variations in the expression of the complement factors in our bladder specimens and that the expressional differences of the complement factors between responders and non-responder at baseline were between a fold-change of only 0.62–1.05. Another theory is that HB02 requires less cellular reprogramming in responders, whereas in non-responders maladaptive reprogramming predominates, resulting in no clinically significant improvement.

### Antigen presentation important biological process in non-responders to HBO2

Proteins associated with antigen processing and presentation were upregulated after HBO2 in non-responders. HBO2 has immune-modulatory effects as demonstrated in many studies. For example, HBO2 may change the polarization of macrophages from a pro-inflammatory M2 phenotype to an anti-inflammatory M1 phenotype [34]. Animal models show that activation of T lymphocytes is dependent on oxygenation and knockout of HIF-1α in T lymphocytes leads to stronger antibacterial effects and survival in septic mice via NF-κB activation [35, 36]. In vivo studies show that HBO2 may increase cytotoxic T lymphocyte infiltration in solid tumors and potentiate PD-1 targeted therapy [35, 37]. We observed that coronin-1 A was upregulated after HBO2. Previous animal studies have reported that coronin-1 A is essential for lymphocyte function [38]. Furthermore, increased RAS protein activator-like 3 (RASAL3) levels were observed. RASAL3 has been implicated in the survival of naïve T lymphocytes [39].

Only considering downregulated proteins, Response to toxic substance and Response to stimuli were biological processes affected by HBO2 in non-responders. The antioxidants SOD-1 and catalase were among proteins affected in non-responders. In line with these findings, we have previously reported that HBO2 reduces oxidative stress and SOD-1 and SOD-2 levels in the irradiated rat urinary bladder [4]. Moreover, lipocalin-1 was among the top DEPs that decreased after HBO2 overall, but showed a numerical, though non-significant, increase in responders. Lipocalin-1 constituted also the top DEP distinguishing the HBO2 response the most in non-responders from responders. Lipocalin-1 is predominantly expressed in glands such as the lacrimal glands and has been shown to function as a scavenger during oxidative stress [40].

### Biological processes affected in the urinary bladder of responders to HBO2

The expression of DEPs among responders was heterogeneous, with fewer significant DEPs compared to non-responders. The greater heterogeneity observed among responders compared with non-responders in our limited sample set may be incidental. Biopsies obtained before and after HB02 were not collected from identical locations within the urinary bladder, and both biopsy depth and tissue composition may therefore vary. Consequently, the relative proportions of urothelial epithelium, lamina propria, and smooth muscle likely differed between samples. In addition, the duration of radiation cystitis prior to HB02 treatment may have influenced the response of the proteome to HB02. Such variability is consistent with well-documented heterogeneity in tissue biopsies analyzed by proteomic approaches as for example described for the prostate gland [41].

As noted, proteins involved in translation were more highly expressed in responders than in non-responders at baseline. In responders, translation-related pathways were among the top enriched biological pathways, with proteins in these pathways downregulated by HBO2. EIFs are important for the initiation of translation [42]. EIF1, EIF5A, EIF5B and EIF4A2 were currently downregulated after HBO2 in responders. It has previously been reported that ionization radiation induces sumoylation of EIF4A2 [43]. During hypoxia the activity of EIF4A2 regulating translation increases in cardiomyocytes [44]. EIF5A has been shown to be important for radioresistance after neoadjuvant treatment of rectal cancer [45]. Studies have shown that oxygen levels regulate the post-translational modification of EIF5A through hypusination, thereby influencing HIF-1α expression in tumour cells [46].

Carbon dioxide transport and hydrogen peroxide catabolic process were enriched biological pathways after HB02 in responders where hemoglobin subunit alpha, hemoglobin subunit gamma-1, hemoglobin subunit delta and hemoglobin subunit beta were upregulated after HBO2. Both carbonic anhydrase 1 and carbonic anhydrase 2 increased in the urinary bladder tissue after HBO2 in responders. High oxygen pressure may potentially induce vasoconstriction in bladder blood vessels. Animal studies show that treatment with the carbonic anhydrase inhibitor acetazolamide prevents oxygen-induced vasoconstriction in the rat cerebral brain [47]. Carbonic anhydrase is present in the lung and catalyzes the reversible hydration of carbon dioxide and is a regulator of pH upregulated in animal models of ischemia-reperfusion-induced acute lung injury [48]. Carbonic anhydrase 2 expression has been detected in urothelial carcinoma, but not in the normal urothelium, and the expression was demonstrated to be higher in muscle-invasive cancer than in non-muscle-invasive cancer [49].

NADH dehydrogenase [ubiquinone] 1 beta subcomplex subunit 8, mitochondrial, (NDUFB8) was increased after HBO2 in responders. Studies show that the expression of NDUFB8 increases in myocytes after exposure to cisplatin in vitro [50]. Among the top downregulated proteins after HBO2 was Ran-binding protein 9 (RANBP9), a scaffold protein present in the cell nucleus and cytoplasm [51]. RANBP9 is involved in stress responses to DNA damage after exposure to ionization radiation, ultraviolet light or chemotherapy [52]. Ras-related protein Rap-1b (RAP1B) was among downregulated proteins after HBO2 in responders. RAP1B has been shown to be important for translocation of NRF2 into the nucleus during hypoxia [53].

### HBO2 effects on neuronal control

While antioxidation and inflammatory processes were important processes in non-responders, these processes did not seem to be of importance for the clinical response to HBO2 in responders. Therefore, other mechanisms could contribute to the clinical effects of HBO2 in the urinary bladder or that HBO2 affects biological processes indirectly altering the function of the urinary bladder. In animal models it has previously been demonstrated that the micturition reflex is affected in radiation cystitis [8]. Therefore, it is possible that HBO2 may affect the neuronal control of the urinary bladder in some patients. This is supported by animal studies showing that HBO2 may be effective against neuronal damage after acute axonal injury and spinal cord injury [54]. HBO2 has also been used in the treatment of patients with spinal cord injury, suggesting that HBO2 may be effective in improving neurological function [55]. To note, there was an absence of morphological changes including signs of inflammation in our bladder specimens, indicating that symptoms of radiation cystitis may arise also from other levels than the urinary bladder [9].

### Limitations

A limitation of the morphological studies is that only very small and superficial biopsies were obtained in this study. Due to the risk of complications, mainly bleeding, larger specimens including the bladder musculature could not be motivated due to ethical considerations. Further, it is important to emphasize that, since biopsies were taken 4–6 months after HBO2 treatment, any changes induced by HBO2 in the urinary bladders of responders may have occurred earlier.

The study is further limited by the small number of samples assessed with proteomics and the heterogeneity among patient samples. For the comparison before and after HB02 in responders a post-hoc cut-off for fold-change was therefore used. Our findings are therefore exploratory and hypothesis-generating and need to be validated in larger cohorts of patients undergoing HB02 due to radiation cystitis. Although immunohistochemistry confirmed the presence of the biomarkers identified with proteomics, it did not validate the between-group differences observed in the proteomic analyses. If the proteomic findings represent true differences, the lack of concordance may reflect differences in detection depth between proteomics and immunohistochemistry, limited antibody sensitivity or sampling variability.

## Conclusions

HBO2 may change multiple biological pathways regulating the immune system, complement activation and translation in radiation cystitis. Changes induced by HBO2 were paradoxically the most prominent in patients not responding to HBO2. The involvement of mast cells in the clinical efficacy of HBO2 in radiation cystitis is suggested. We are currently in the process of initiating a study to validate our biomarker findings in a larger cohort of patients with radiation cystitis treated with HBO2.​ ​

## Supplementary information

Below is the link to the electronic supplementary material.

Supplementary Methods

Supplementary Figure 1

Supplementary Figure 2

Supplementary Figure 3

Supplementary Figure 4

Supplementary Figure 5

Supplementary Figure 6

Supplementary Figure 7

Supplementary Figure 8

Supplementary Figure 9

Supplementary Tables 1-14

## Data Availability

The MS proteomics data have been deposited to the ProteomeXchange Consortium (http://proteomecentral.proteomexchange.org) via the PRIDE partner repository with the data set identifier PXD085707. Requests for additional data can be sent to the Institute of Clinical Sciences at the Sahlgrenska Academy, University of Gothenburg, Medicinaregatan 3 A SE-413 90 Göteborg, Sweden. Email: klinvet@gu.se.

## Abbreviations

CPA3: Carboxypeptidase A
DEPs: differentially expressed proteins
EPIC: Expanded Prostate Cancer Index Composite
HBO2: hyperbaric oxygen
PCA: principal component analysis
SOD-1: superoxide dismutase-1

## References

[1] Sammour T, Kahokehr AA. Pelvic Radiation Disease. Clin Colon Rectal Surg. 2022;35(3):204–11. 10.1055/s-0041-1740041 PMC9374535 35966384

[2] Lonergan PE, Baskin A, Greenberg SA, Mohamad O, Washington SL 3rd, Zhao S, Cowan JE, Broering JM, Nguyen HG, Cooperberg MR, et al. The Long-term Incidence and Quality of Life Outcomes Associated With Treatment-Related Toxicities of External Beam Radiotherapy for Prostate Cancer. Urology. 2023;178:114–9. 10.1016/j.urology.2023.05.012 37244430

[3] Stewart FA. Mechanism of bladder damage and repair after treatment with radiation and cytostatic drugs. Br J Cancer Suppl. 1986;7:280–91. PMC2149791 3521706

[4] Oscarsson N, Ny L, Molne J, Lind F, Ricksten SE, Seeman-Lodding H, Giglio D. Hyperbaric oxygen treatment reverses radiation induced pro-fibrotic and oxidative stress responses in a rat model. Free Radic Biol Med. 2017;103:248–55. 10.1016/j.freeradbiomed.2016.12.036 28034833

[5] Romanenko A, Morimura K, Wanibuchi H, Wei M, Zaparin W, Vinnichenko W, Kinoshita A, Vozianov A, Fukushima S. Urinary bladder lesions induced by persistent chronic low-dose ionizing radiation. Cancer Sci. 2003;94(4):328–33. 10.1111/j.1349-7006.2003.tb01441.x PMC11160273 12824899

[6] Vera PL, Preston DM, Moldwin RM, Erickson DR, Mowlazadeh B, Ma F, Kouzoukas DE, Meyer-Siegler KL, Fall M. Elevated Urine Levels of Macrophage Migration Inhibitory Factor in Inflammatory Bladder Conditions: A Potential Biomarker for a Subgroup of Interstitial Cystitis/Bladder Pain Syndrome Patients. Urology. 2018;116:55–62. 10.1016/j.urology.2018.02.039 PMC5975106 29580781

[7] Giglio D, Wasen C, Molne J, Suchy D, Swanpalmer J, Jabonero Valbuena J, Tobin G, Ny L. Downregulation of toll-like receptor 4 and IL-6 following irradiation of the rat urinary bladder. Clin Exp Pharmacol Physiol. 2016;43(7):698–705. 10.1111/1440-1681.12583 27117224

[8] Giglio D, Podmolikova L, Tobin G. Changes in the Neuronal Control of the Urinary Bladder in a Model of Radiation Cystitis. J Pharmacol Exp Ther. 2018;365(2):327–35. 10.1124/jpet.117.246371 29530925

[9] Oscarsson N, Muller B, Rosen A, Lodding P, Molne J, Giglio D, Hjelle KM, Vaagbo G, Hyldegaard O, Vangedal M, et al. Radiation-induced cystitis treated with hyperbaric oxygen therapy (RICH-ART): a randomised, controlled, phase 2–3 trial. Lancet Oncol. 2019;20(11):1602–14. 10.1016/S1470-2045(19)30494-2 31537473

[10] Svalestad J, Hellem S, Thorsen E, Johannessen AC. Effect of hyperbaric oxygen treatment on irradiated oral mucosa: microvessel density. Int J Oral Maxillofac Surg. 2015;44(3):301–7. 10.1016/j.ijom.2014.12.012 25604154

[11] Spiegelberg L, Braks JA, Groeneveldt LC, Djasim UM, van der Wal KG, Wolvius EB. Hyperbaric oxygen therapy as a prevention modality for radiation damage in the mandibles of mice. J Craniomaxillofac Surg. 2015;43(2):214–9. 10.1016/j.jcms.2014.11.008 25523397

[12] Dressler FF, Diedrichs F, Sabtan D, Hinrichs S, Krisp C, Gemoll T, Hennig M, Mackedanz P, Schlotfeldt M, Voss H, et al. Proteomic analysis of the urothelial cancer landscape. Nat Commun. 2024;15(1):4513. 10.1038/s41467-024-48096-5 PMC11130393 38802361

[13] Chang Q, Chen Y, Yin J, Wang T, Dai Y, Wu Z, Guo Y, Wang L, Zhao Y, Yuan H, et al. Comprehensive Urinary Proteome Profiling Analysis Identifies Diagnosis and Relapse Surveillance Biomarkers for Bladder Cancer. J Proteome Res. 2024;23(6):2241–52. 10.1021/acs.jproteome.4c00199 38787199

[14] Huang S, Wang X, Sun Y, Lu X, Jiang W, Chen Z, Huang Y, Chi P. TMT-labelled quantitative proteomic analysis to identify the proteins underlying radiation-induced colorectal fibrosis in rats. J Proteom. 2020;223:103801. 10.1016/j.jprot.2020.103801 32376500

[15] Zwaans BMM, Bartolone SN, Chancellor MB, Nicolai HE, Lamb LE. Altered Angiogenic Growth Factors in Urine of Prostate Cancer Survivors With Radiation History and Radiation Cystitis. Urology. 2018;120:180–6. 10.1016/j.urology.2018.07.025 PMC6589609 30059715

[16] Gulli F, Geddes TJ, Pruetz BL, Wilson GD. Investigation of the physiological response of radiation-induced cystitis patients using hyperbaric oxygen. Clin Transl Radiat Oncol. 2023;38:104–10. 10.1016/j.ctro.2022.10.009 PMC9672132 36407487

[17] Perez-Riverol Y, Bai J, Bandla C, Garcia-Seisdedos D, Hewapathirana S, Kamatchinathan S, Kundu DJ, Prakash A, Frericks-Zipper A, Eisenacher M, et al. The PRIDE database resources in 2022: a hub for mass spectrometry-based proteomics evidences. Nucleic Acids Res. 2022;50(D1):D543–52. 10.1093/nar/gkab1038 PMC8728295 34723319

[18] Brook M, Tomlinson GH, Miles K, Smith RW, Rossi AG, Hiemstra PS, van ‘t Wout EF, Dean JL, Gray NK, Lu W, et al. Neutrophil-derived alpha defensins control inflammation by inhibiting macrophage mRNA translation. Proc Natl Acad Sci U S A. 2016;113(16):4350–5. 10.1073/pnas.1601831113 PMC4843457 27044108

[19] Fu J, Zong X, Jin M, Min J, Wang F, Wang Y. Mechanisms and regulation of defensins in host defense. Signal Transduct Target Ther. 2023;8(1):300. 10.1038/s41392-023-01553-x PMC10423725 37574471

[20] Ventura TMO, Ribeiro NR, Taira EA, de Souza ESCM, Rubira CMF, Santos P, Buzalaf MAR. Radiotherapy changes acquired enamel pellicle proteome in head and neck cancer patients. J Dent. 2021;108:103642. 10.1016/j.jdent.2021.103642 33757866

[21] Cheng L, Lei X, Yang Z, Kong Y, Xu P, Peng S, Wang J, Chen C, Dong Y, Hu X, et al. Histatin 1 enhanced the speed and quality of wound healing through regulating the behaviour of fibroblast. Cell Prolif. 2021;54(8):e13087. 10.1111/cpr.13087 PMC8349656 34255393

[22] Matsuda K, Okamoto N, Kondo M, Arkwright PD, Karasawa K, Ishizaka S, Yokota S, Matsuda A, Jung K, Oida K, et al. Mast cell hyperactivity underpins the development of oxygen-induced retinopathy. J Clin Invest. 2017;127(11):3987–4000. 10.1172/JCI89893 PMC5663365 28990934

[23] Takahashi N, Kuwaki T, Kiyonaka S, Numata T, Kozai D, Mizuno Y, Yamamoto S, Naito S, Knevels E, Carmeliet P, et al. TRPA1 underlies a sensing mechanism for O2. Nat Chem Biol. 2011;7(10):701–11. 10.1038/nchembio.640 21873995

[24] Brock TG, Di Giulio C. Prolonged exposure to hyperoxia increases perivascular mast cells in rat lungs. J Histochem Cytochem. 2006;54(11):1239–46. 10.1369/jhc.6A7007.2006 16899761

[25] Riekki R, Harvima IT, Jukkola A, Risteli J, Oikarinen A. The production of collagen and the activity of mast-cell chymase increase in human skin after irradiation therapy. Exp Dermatol. 2004;13(6):364–71. 10.1111/j.0906-6705.2004.00164.x 15186323

[26] Sedgwick DM, Ferguson A. Dose-response studies of depletion and repopulation of rat intestinal mucosal mast cells after irradiation. Int J Radiat Biol. 1994;65(4):483–95. 10.1080/09553009414550561 7908936

[27] Malik ST, Birch BR, Voegeli D, Fader M, Foria V, Cooper AJ, Walls AF, Lwaleed BA. Distribution of mast cell subtypes in interstitial cystitis: implications for novel diagnostic and therapeutic strategies? J Clin Pathol. 2018;71(9):840–4. 10.1136/jclinpath-2017-204881 29764932

[28] Aronsson P, Johnsson M, Vesela R, Winder M, Tobin G. Adenosine receptor antagonism suppresses functional and histological inflammatory changes in the rat urinary bladder. Auton Neurosci. 2012;171(1–2):49–57. 10.1016/j.autneu.2012.10.006 23142515

[29] Kalm M, Andreasson U, Bjork-Eriksson T, Zetterberg H, Pekny M, Blennow K, Pekna M, Blomgren K. C3 deficiency ameliorates the negative effects of irradiation of the young brain on hippocampal development and learning. Oncotarget. 2016;7(15):19382–94. 10.18632/oncotarget.8400 PMC4991390 27029069

[30] Park JY, Loh S, Cho EH, Choi HJ, Na TY, Nemeno JG, Lee JI, Yoon TJ, Choi IS, Lee M, et al. SIGN-R1 and complement factors are involved in the systemic clearance of radiation-induced apoptotic cells in whole-body irradiated mice. Biochem Biophys Res Commun. 2015;463(4):1064–70. 10.1016/j.bbrc.2015.06.059 26079881

[31] Surace L, Lysenko V, Fontana AO, Cecconi V, Janssen H, Bicvic A, Okoniewski M, Pruschy M, Dummer R, Neefjes J, et al. Complement is a central mediator of radiotherapy-induced tumor-specific immunity and clinical response. Immunity. 2015;42(4):767–77. 10.1016/j.immuni.2015.03.009 25888260

[32] Frandsen B, Lose G, Holm-Bentzen M. Autorosette inhibition factor: a positive acute phase reactant in interstitial cystitis. Eur Urol. 1988;14(4):309–12. 10.1159/000472967 2458935

[33] Wu KY, Cao B, Wang CX, Yang XL, Zhao SJ, Diao TY, Lin LR, Zhao GX, Zhou W, Yang JR, et al. The C5a/C5aR1 Axis Contributes to the Pathogenesis of Acute Cystitis Through Enhancement of Adhesion and Colonization of Uropathogenic E. coli. Front Cell Infect Microbiol. 2022;12:824505. 10.3389/fcimb.2022.824505 PMC9005882 35433513

[34] Yuen CM, Tsai HP, Tseng TT, Tseng YL, Lieu AS, Kwan AL, Chang AYW. Hyperbaric Oxygen Therapy as a Novel Approach to Modulating Macrophage Polarization for the Treatment of Glioblastoma. Biomedicines. 2024;12(7):1383. 10.3390/biomedicines12071383 PMC11274314 39061957

[35] Ohta A, Diwanji R, Kini R, Subramanian M, Ohta A, Sitkovsky M. In vivo T cell activation in lymphoid tissues is inhibited in the oxygen-poor microenvironment. Front Immunol. 2011;2:27. 10.3389/fimmu.2011.00027 PMC3342240 22566817

[36] Thiel M, Caldwell CC, Kreth S, Kuboki S, Chen P, Smith P, Ohta A, Lentsch AB, Lukashev D, Sitkovsky MV. Targeted deletion of HIF-1alpha gene in T cells prevents their inhibition in hypoxic inflamed tissues and improves septic mice survival. PLoS ONE. 2007;2(9):e853. 10.1371/journal.pone.0000853 PMC1959117 17786224

[37] Liu X, Ye N, Liu S, Guan J, Deng Q, Zhang Z, Xiao C, Ding ZY, Zhang BX, Chen XP, et al. Hyperbaric Oxygen Boosts PD-1 Antibody Delivery and T Cell Infiltration for Augmented Immune Responses Against Solid Tumors. Adv Sci (Weinh). 2021;8(15):e2100233. 10.1002/advs.202100233 PMC8336507 34085419

[38] Foger N, Rangell L, Danilenko DM, Chan AC. Requirement for coronin 1 in T lymphocyte trafficking and cellular homeostasis. Science. 2006;313(5788):839–42. 10.1126/science.1130563 16902139

[39] Muro R, Nitta T, Kitajima M, Okada T, Suzuki H. Rasal3-mediated T cell survival is essential for inflammatory responses. Biochem Biophys Res Commun. 2018;496(1):25–30. 10.1016/j.bbrc.2017.12.159 29291408

[40] Lechner M, Wojnar P, Redl B. Human tear lipocalin acts as an oxidative-stress-induced scavenger of potentially harmful lipid peroxidation products in a cell culture system. Biochem J. 2001;356(Pt 1):129–35. 10.1042/0264-6021:3560129 PMC1221820 11336644

[41] Guo T, Li L, Zhong Q, Rupp NJ, Charmpi K, Wong CE, Wagner U, Rueschoff JH, Jochum W, Fankhauser CD, et al. Multi-region proteome analysis quantifies spatial heterogeneity of prostate tissue biomarkers. Life Sci Alliance. 2018;1(2):e201800042. 10.26508/lsa.201800042 PMC6078179 30090875

[42] Jackson RJ, Hellen CU, Pestova TV. The mechanism of eukaryotic translation initiation and principles of its regulation. Nat Rev Mol Cell Biol. 2010;11(2):113–27. 10.1038/nrm2838 PMC4461372 20094052

[43] Jongjitwimol J, Baldock RA, Morley SJ, Watts FZ. Sumoylation of eIF4A2 affects stress granule formation. J Cell Sci. 2016;129(12):2407–15. 10.1242/jcs.184614 PMC4920252 27160682

[44] Ye F, Wang X, Tu S, Zeng L, Deng X, Luo W, Zhang Z. The effects of NCBP3 on METTL3-mediated m6A RNA methylation to enhance translation process in hypoxic cardiomyocytes. J Cell Mol Med. 2021;25(18):8920–8. 10.1111/jcmm.16852 PMC8435433 34382339

[45] Zhong Y, Chen X, Wu S, Fang H, Hong L, Shao L, Wang L, Wu J. Deciphering colorectal cancer radioresistance and immune microrenvironment: unraveling the role of EIF5A through single-cell RNA sequencing and machine learning. Front Immunol. 2024;15:1466226. 10.3389/fimmu.2024.1466226 PMC11405197 39290702

[46] Tariq M, Ito A, Ishfaq M, Bradshaw E, Yoshida M. Eukaryotic translation initiation factor 5A (eIF5A) is essential for HIF-1alpha activation in hypoxia. Biochem Biophys Res Commun. 2016;470(2):417–24. 10.1016/j.bbrc.2016.01.024 26773503

[47] Gutsaeva DR, Moskvin AN, Zhilyaev SY, Kostkin VB, Demchenko IT. The roles of nitric oxide and carbon dioxide gas in the neurotoxic actions of oxygen under pressure. Neurosci Behav Physiol. 2005;35(7):751–6. 10.1007/s11055-005-0119-9 16433071

[48] Lan CC, Peng CK, Tang SE, Huang KL, Wu CP. Carbonic anhydrase inhibitor attenuates ischemia-reperfusion induced acute lung injury. PLoS ONE. 2017;12(6):e0179822. 10.1371/journal.pone.0179822 PMC5482472 28644844

[49] Tachibana H, Gi M, Kato M, Yamano S, Fujioka M, Kakehashi A, Hirayama Y, Koyama Y, Tamada S, Nakatani T, et al. Carbonic anhydrase 2 is a novel invasion-associated factor in urinary bladder cancers. Cancer Sci. 2017;108(3):331–7. 10.1111/cas.13143 PMC5378286 28004470

[50] Le Bacquer O, Sanchez P, Patrac V, Rivoirard C, Saroul N, Giraudet C, Kocer A, Walrand S. Cannabidiol protects C2C12 myotubes against cisplatin-induced atrophy by regulating oxidative stress. Am J Physiol Cell Physiol. 2024;326(4):C1226–36. 10.1152/ajpcell.00622.2023 38406827

[51] Suresh B, Ramakrishna S, Baek KH. Diverse roles of the scaffolding protein RanBPM. Drug Discov Today. 2012;17(7–8):379–87. 10.1016/j.drudis.2011.10.030 22094242

[52] Palmieri D, Tessari A, Coppola V. Scorpins in the DNA Damage Response. Int J Mol Sci. 2018;19(6):1794. 10.3390/ijms19061794 PMC6032341 29914204

[53] Yang Y, Wang X, Zhang J, Gu H, Zhang S, Sun H, Liu J, Fan R. Abnormal phenotype of Nrf2 is associated with poor prognosis through hypoxic/VEGF-A-Rap1b/VEGFR2 pathway in gastric cancer. Aging. 2022;14(7):3293–312. 10.18632/aging.204013 PMC9037254 35417854

[54] Sri Dewi Untari NK, Kusumastuti K, Suryokusumo G, Sudiana IK. Protective Effect of Hyperbaric Oxygen Treatment on Axon Degeneration after Acute Motor Axonal Neuropathy. *Autoimmune Dis* 2021, 2021:6627779. 10.1155/2021/6627779 PMC8592739 34790416

[55] Siglioccolo A, Gammaldi R, Vicinanza V, Galardo A, Caterino V, Palmese S, Ferraiuoli C, Calicchio A, Romanelli A. Advance in hyperbaric oxygen therapy in spinal cord injury. Chin J Traumatol. 2024;27(6):348–53. 10.1016/j.cjtee.2023.05.002 PMC11624306 37271686

