# Supplementary material for: Candidate biomarkers predicting clinical outcome after hyperbaric oxygen against chronic radiation cystitis
Source: BMC Cancer. 2026 Nov 10;26:1411. doi: 10.1186/s12885-026-17309-z (PMC13760933; doi:10.1186/s12885-026-17309-z)
Supplement: Supplementary file 1 [file 12885_2026_17309_MOESM1_ESM.docx]

**Global protein quantification**

***Proteomic sample preparation***

Relative quantification was performed to compare protein expression in deparaffinized formalin-fixed paraffin-embedded urinary biopsies (20 sections à 10 µm). Proteins were extracted in lysis buffer (2% sodium dodecyl sulfate (SDS), 50 mM triethylammonium bicarbonate (TEAB)) using a FastPrep^®^-24 instrument (Matrix D, MP Biomedicals, Irvine, CA, USA). Samples were reduced (DTT, 100 mM, 60°C, 30 min) and processed with a modified filter-aided sample preparation (FASP) method. In short, samples were concentrated and washed with 8M urea on Microcon-30kDa centrifugal filters (Merck, XXX, XXX) and alkylated (MMTS, 10 mM, 30 min in room temperature). Samples were digested with trypsin (0.3 µg, Pierce MS grade Trypsin, Thermo Fisher Scientific, Waltham, MA, USA, 37°C, overnight) in digestion buffer (0.5 % sodium deoxycholate (SDC), 50 mM TEAB). Peptides were isobaric labelled using Tandem Mass Tag (TMTpro, 16-plex) reagents (Thermo Fischer Scientific) according to the manufacturer’s instructions. The labelled samples were pooled into one TMT-set, SDC was removed by acidification (10% trifluoroacetic acid (TFA)) and further purified using Pierce peptide desalting spin columns (Thermo Scientific), according to the manufacturer´s instructions. The TMT-sets were fractionated by basic reversed-phase chromatography on a Dionex Ultimate 3000 UPLC system (Thermo Fisher Scientific). Peptide samples were labelled using TMTpro 16-plex isobaric mass tagging reagents (Thermo Fisher Scientific). The labelled samples were pooled into one TMT-set and purified using Pierce peptide desalting spin columns (Thermo Scientific), according to the manufacturer´s instructions. The TMT-set was fractionated by basic reversed-phase chromatography on a Dionex Ultimate 3000 UPLC system (Thermo Fisher Scientific). Peptide separations were performed using a reversed-phase XBridge BEH C18 column (3.5 μm, 3.5x150 mm, Waters Corporation) and a stepped gradient from 3% to 90% acetonitrile in 10 mM ammonium formate buffer (pH 10) over 30 min. 96 primary fractions were combined to 20 final fractions which were evaporated and reconstituted in 3% acetonitrile, 0.1% trifluoroacetic for LC-MS3 analysis.

***LC-MS3 analysis***

The fractions were analyzed twice on an Orbitrap Fusion Tribrid mass spectrometer interfaced with an Easy nanoLC 1200 liquid chromatography system (Thermo Fisher Scientific). Peptides were trapped on an Acclaim Pepmap 100 C18 trap column (100 μm x 2 cm, particle size 5 μm, Thermo Fisher Scientific) and separated on an in-house packed analytical column (35 cm x 75 μm, particle size 3 μm, Reprosil-Pur C18, Dr. Maisch) using a stepped gradient from 5% to 80% acetonitrile in 0.2% formic acid over 90 min at a flow of 300 nL/min. The precursor ion mass spectra were acquired at a resolution of 120 000 and an m/z range of 375-1500. Using a cycle time of 1.5 seconds the most abundant precursors with charges 2–7 were isolated with an m/z window of 0.7 and fragmented by collision induced dissociation (CID) at 30%. Fragment spectra were recorded in the ion trap at Rapid scan rate. Dynamic exclusion was set to 45 sec. The ten most abundant MS2 fragment ions were isolated using multi-notch isolation for further MS3 fragmentation. MS3 fragmentation was performed using higher-energy collision dissociation (HCD) at 55% and the MS3 spectra were recorded in the Orbitrap at 50 000 resolution and an m/z range of 100–500.

***Database matching and quantification 2021***

Raw files were processed and analysed with Proteome Discoverer (ver 2.4, Thermo Fisher Scientific) The data was matched against *Human* SwissProt database (20.361 entries, Jan 2021) using Mascot 2.5.1 (Matrix Science, London, United Kingdom) as a search engine with a precursor tolerance of 5 ppm and a fragment ion tolerance of 0.6 Da. Tryptic peptides were accepted with 0 missed cleavage. Methionine oxidation was set as a variable modifications and cysteine alkylation, TMTpro on lysine and peptide N-termini were set as fixed modifications. Percolator was used for PSM validation with a strict FDR threshold of 1%. For quantification TMT reporter ions were identified in the MS3 HCD spectra with 3 mmu mass tolerance and the TMT reporter intensity values for each sample were normalized on the total peptide amount. The SPS threshold was set to 65%. Only unique peptides were used for relative quantification and proteins were filtered at FDR of 1%.

| **PCF name** | **ID** | **Group** | **Group sample ID** |
| --- | --- | --- | --- |
| C1 | 51_1 | Nonresponder = control | nonResp1:1 |
| C2 | 51_2 | Nonresponder = control | nonResp1:2 |
| C3 | 53_1 | Nonresponder = control | nonResp2:1 |
| C4 | 53_4 | Nonresponder = control | nonResp2:2 |
| C5 | 100_1 | Nonresponder = control | nonResp3:1 |
| C6 | 100_4 | Nonresponder = control | nonResp3:2 |
| C7 | 105_1 | Nonresponder = control | nonResp4:1 |
| C8 | 105_2 | Nonresponder = control | nonResp4:2 |
| R1 | 28_1 | Responder | Resp1:1 |
| R2 | 28_2 | Responder | Resp1:2 |
| R3 | 34_1 | Responder | Resp2:1 |
| R4 | 34_4 | Responder | Resp2:2 |
| R5 | 98_1 | Responder | Resp3:1 |
| R6 | 98_4 | Responder | Resp3:2 |
| R7 | 124_1 | Responder | Resp3:1 |
| R8 | 124_4 | Responder | Resp3:2 |

**Study design and TMT-tag**

| **Tag** | **PCF name** | **ID** | **Group sample ID** |
| --- | --- | --- | --- |
| 126 | C1 | 51_1 | nonResp1:1 |
| 127N | R1 | 28_1 | Resp1:1 |
| 127C | C2 | 51_2 | nonResp1:2 |
| 128N | R2 | 28_2 | Resp1:2 |
| 128C | C3 | 53_1 | nonResp2:1 |
| 129N | R3 | 34_1 | Resp2:1 |
| 129C | C4 | 53_4 | nonResp2:2 |
| 130N | R4 | 34_4 | Resp2:2 |
| 130C | C5 | 100_1 | nonResp3:1 |
| 131N | R5 | 98_1 | Resp3:1 |
| 131C | C6 | 100_4 | nonResp3:2 |
| 132N | R6 | 98_4 | Resp3:2 |
| 132C | C7 | 105_1 | nonResp4:1 |
| 133N | R7 | 124_1 | Resp3:1 |
| 133C | C8 | 105_2 | nonResp4:2 |
| 134N | R8 | 124_4 | Resp3:2 |
